# Identification of Key Genes and Pathways Associated With Paclitaxel Resistance in Esophageal Squamous Cell Carcinoma Based on Bioinformatics Analysis

**DOI:** 10.3389/fgene.2021.671639

**Published:** 2021-08-11

**Authors:** Zhimin Shen, Mingduan Chen, Fei Luo, Hui Xu, Peipei Zhang, Jihong Lin, Mingqiang Kang

**Affiliations:** ^1^Department of Thoracic Surgery, Fujian Medical University Union Hospital, Fuzhou, China; ^2^Key Laboratory of Ministry of Education for Gastrointestinal Cancer, Fujian Medical University, Fuzhou, China; ^3^Fujian Key Laboratory of Tumor Microbiology, Fujian Medical University, Fuzhou, China; ^4^Fujian Key Laboratory of Cardio-Thoracic Surgery, Fujian Medical University, Fuzhou, China

**Keywords:** esophageal squamous cell carcinoma, paclitaxel resistance, TCGA database, GSE data, bioinformatics analysis

## Abstract

Esophageal squamous cell carcinoma (ESCC) ranks as the fourth leading cause of cancer-related death in China. Although paclitaxel has been shown to be effective in treating ESCC, the prolonged use of this chemical will lead to paclitaxel resistance. In order to uncover genes and pathways driving paclitaxel resistance in the progression of ESCC, bioinformatics analyses were performed based on The Cancer Genome Atlas (TCGA) database and the Gene Expression Omnibus (GEO) database including GSE86099 and GSE161533. Differential expression analysis was performed in TCGA data and two GEO datasets to obtain differentially expressed genes (DEGs). Based on GSE161533, weighted gene co-expression network analysis (WGCNA) was conducted to identify the key modules associated with ESCC tumor status. The DEGs common to the two GEO datasets and the genes in the key modules were intersected to obtain the paclitaxel resistance-specific or non-paclitaxel resistance-specific genes, which were subjected to subsequent least absolute shrinkage and selection operator (LASSO) feature selection, whereby paclitaxel resistance-specific or non-paclitaxel resistance-specific key genes were selected. Ten machine learning models were used to validate the biological significance of these key genes; the potential therapeutic drugs for paclitaxel resistance-specific genes were also predicted. As a result, we identified 24 paclitaxel resistance-specific genes and 18 non-paclitaxel resistance-specific genes. The ESCC machine classifiers based on the key genes achieved a relatively high AUC value in the cross-validation and in an independent test set, GSE164158. A total of 207 drugs (such as bevacizumab) were predicted to be alternative therapeutics for ESCC patients with paclitaxel resistance. These results might shed light on the in-depth research of paclitaxel resistance in the context of ESCC progression.

## Introduction

Esophageal squamous cell carcinoma (ESCC) is one of the most lethal malignancies in the world and occurs with an especially high frequency in China. As the fourth leading cause of cancer-related deaths, ESCC kills about 250,000 people in China every year (Peng et al., 2018). The global incidence burden and mortality have been increasing over time (Batra et al., 2019). The disease is aggressive with poor overall survival and is generally unresectable. Therefore, it is urgent to develop effective therapeutic strategies against ESCC.

Although most ESCC patients receive standard treatments including surgery, radiotherapy, and chemotherapy, the long-term outcomes for these patients remain dismal, with a 5-year survival rate of around 30% (Liu et al., 2012). The poor prognostic outcomes result from the failure of early diagnosis and acquired chemoresistance. The targeted therapies and immunotherapies approved by the US Food and Drug Administration (FDA) only resulted in significant improvements in survival for a few specific subgroups of patients who are positive for certain biomarkers (Lam and Kwong, 2018; Ma et al., 2018; Barsouk et al., 2019). The remaining majority of patients without such biomarkers still rely on traditional chemotherapy and radiation therapy. Paclitaxel-based regimens have been examined and reported to be effective in multiple clinical trials (Hirano and Kato, 2019). However, prolonged therapeutic management will inevitably lead to the development of paclitaxel resistance, the predominant cause of treatment failure, which poses a challenge to ESCC treatment. Thus, there is an intense focus on how to counteract paclitaxel resistance, especially the underlying molecular mechanisms.

High-throughput technologies have been broadly employed in cancer research, and large amounts of data are being created from various microarrays, tissue arrays, and next-generation sequencing platforms. Bioinformatics and computational biology are powerful tools to analyze massive data. To identify the genes responsible for paclitaxel resistance, we identified the differentially expressed genes (DEGs) between paclitaxel-resistant embryo/cancer sequence A (ECSA) cell lines and their parental cell lines based on the GSE86099 dataset. We also downloaded the mRNA expression matrix data of ESCC from The Cancer Genome Atlas (TCGA) and the Gene Expression Omnibus (GEO) database to analyze the DEGs between normal and tumor tissues. Afterward, weighted gene co-expression network analysis (WGCNA) was performed to screen the modules associated with ESCC tumor status. In addition, we evaluated the prognostic potential of ESCC-specific genes associated with paclitaxel resistance using the least absolute shrinkage and selection operator (LASSO) and machine learning, which provides new insights into paclitaxel resistance and potential targets for overcoming resistance in ESCC.

## Materials and Methods

### Data Collection

GSE86099 was obtained from the GEO database (http://www.ncbi.nlm.nih.gov/geo/). This dataset was from a study conducted by Wang et al. (2016), which consisted of four paclitaxel-resistant ECSA cell lines and four parental cell lines (non-paclitaxel-resistant). The keywords “Esophageal” and “*Homo sapiens*” were used as query to search ESCC-associated datasets from the GEO database. The GEO datasets used in this study met the following criteria: (1) the dataset contains both ESCC samples and control samples; (2) each sample was assigned a group label; (3) the type of platform was restricted to “microarray”; (4) the gene symbol or GeneBank ID was available for each probe; (5) the patient was not previously treated with chemotherapy or non-paclitaxel drugs; and 6) the number of samples in the dataset was larger than 10. Finally, we obtained GSE161533 from the GEO database, which included 28 ESCC samples and 28 normal samples. Gene expression and subtype data of the esophageal carcinoma samples were downloaded from the TCGA database (https://www.cancer.gov/about-nci/organization/ccg/research/structural-genomics/tcga), which included 164 esophageal carcinoma samples and 11 normal samples. An independent dataset (GSE164158) was used for the validation of our current findings based on a machine learning classifier.

### Identification of DEGs and Construction of Co-expression Network

The mean value of gene expression was retained when the gene symbol mapped with multiple probes and the genes with missing value or with zero value were excluded. The R “limma” package was used to perform differential expression analysis and data normalization. Data scaling of GSE86099, GSE161533, and TCGA Esophageal Carcinoma (TCGA-ESCA) was implemented by logarithmic conversion in R. DEGs were identified with the threshold of |log2FoldChange| > 0.263 and *p*_adj_. < 0.05 (adjusted *p*-value). The top 25 most significant upregulated and downregulated DEGs (sorted by the |log2FoldChange|) were extracted and visualized in a heatmap using the R “pheatmap” package. The corresponding volcano plot was visualized by R “ggpubr” (https://cran.r-project.org/web/packages/ggpubr/index.html) and “ggthemes” (https://cran.r-project.org/web/packages/ggthemes/index.html) packages. The top 10 upregulated and downregulated DEGs (ranked by *p*_adj._ value) were labeled with gene symbols in the volcano plots.

The R “WGCNA” package was used to construct a scale-free topological matrix based on GSE161533, which included 22,880 genes and 56 samples. The pickSoftThreshold function was used to select a suitable power to construct a co-expression network that conforms to the scale-free distribution. Pearson's correlation coefficient (PCC) was used to analyze the correlation between the ESCC tumor status and module eigengene (MM), and the modules with the highest correlations (positive and negative) with the ESCC tumor status were selected as ESCC-specific modules.

### Identification of Paclitaxel Resistance-Specific/Non-paclitaxel Resistance-Specific Genes

Paclitaxel resistance-specific and non-paclitaxel resistance-specific genes were obtained from the intersection among the DEGs in the GSE86099, GSE161533, and the ESCC-specific modules. Specifically, genes that were positively associated with ESCC and upregulated in the ESCC/paclitaxel resistance cell lines by comparing normal and non-paclitaxel resistance cell lines were considered to be responsible for ESCC progression and paclitaxel resistance and were defined as paclitaxel resistance-specific genes. In contrast, genes that were inversely associated with ESCC and downregulated in the ESCC and paclitaxel resistance cell lines by comparing normal and non-paclitaxel resistance cell lines, which showed a low risk of paclitaxel resistance, were defined as non-paclitaxel resistance-specific genes. The correlations between the genes of the ESCC-specific modules and the DEGs from GSE86099 and GSE161533 were visualized by the R “UpSetR” package.

### Identification of Key Genes Using LASSO

The least absolute shrinkage and selection operator (LASSO) was adopted to identify the important features of paclitaxel resistance and non-paclitaxel resistance. The R “glmnet” (https://glmnet.stanford.edu) package was used to perform the LASSO selection with 10-fold cross-validation. The key genes (associated with paclitaxel resistance and non-paclitaxel resistance, including paclitaxel resistance-specific and non-paclitaxel resistance-specific key genes) were identified based on the coefficient weight.

TCGA-ESCA was used to display the expression levels of the key genes across the different ESCC tumor stages. Statistical significance was analyzed using analysis of variance (ANOVA) with a Python script. The R “limma” package was used to analyze the expression levels of the key genes in the ESCC tumor group compared with the normal group. The R “beanplot” and R “boxplot” were used to visualize the expression levels of the key genes in the TCGA-ESCA dataset.

### Machine Learning-Based Validation of the Key Genes

To further uncover the prognostic value and biological significance of the key genes, we constructed ESCC classifiers using 10 machine learning algorithms. Firstly, we split GSE161533 into a training set and a validation set with a 7:3 ratio. Ten machine classifiers (SVM, random forest, ExtraTree, AdaBoost, GradientBoosting, MLP, KNeighbors, logistic regression, linear discriminant analysis, and GaussianNB) were performed on the training set; the generalization performance of these models was validated in both validation and test sets. A 10-fold cross-validation method was used to select the hyperparameters and to avoid overfitting. Then, the ESCC machine classifiers were constructed using a machine learning model from the Python scikit-learn library (Pedregosa et al., 2011). The random state of the classifiers was set as 42 and the mean area under the receiver operating characteristic (ROC) curve (AUC) was calculated after cross-validation. An additional dataset, GSE67269, was used as a test set for the back-propagation neural network (BPNN)-based validation following the same procedure.

### PPI Network Construction

To uncover the interaction between the paclitaxel resistance-specific genes/non-paclitaxel resistance-specific genes, protein–protein interaction (PPI) analysis was performed. Twenty-four paclitaxel resistance-specific genes and 18 non-paclitaxel resistance-specific genes were uploaded onto the Search Tool to obtain the interaction information of their coded proteins. Herein, “*Homo sapiens*” was used to filter the results. Protein interactions with low confidence (a combined score >0.4) were considered acceptable. Based on these criteria, the resulting ^*^TSV file was downloaded from STRING and visualized locally as a PPI network using Cytoscape 3.4.0 software. Nodes without any connection to other nodes were removed from the PPI network. The hub genes in the PPI network were defined according to the degree centrality.

To explore the prognostic role of the hub genes obtained in the PPI networks, we performed Kaplan–Meier survival analysis of the hub genes based on TCGA-ESCA by GEPIA (http://gepia.cancer-pku.cn/). The overall survival (OS) and disease-free survival (DFS) of the hub genes in ESCC were analyzed.

### Functional Enrichment Analysis

The R “clusterProfiler” package was used to investigate the biological function of the genes in this study. The *p*_adj._ value was used to rank the Gene Ontology (GO) terms and pathways, and the pathways with *p*_adj._ < 0.05 were considered significant. The visualization of the top 10 GO terms under the biological process, cellular component, and molecular function branches and the top 10 Kyoto Encyclopedia of Genes and Genomes (KEGG) pathways were implemented by the R “ggplot2” package.

### Drug Interaction Prediction for Paclitaxel Resistance-Specific Key Genes

The Drug Gene Interaction Database (DGIdb; www.dgidb.org) was used to predict the interaction between genes and drugs. We uploaded the paclitaxel resistance-specific key genes onto the DGIdb to obtain the potential targeted drugs effective for paclitaxel resistance in ESCC.

## Results

### Screening of DEGs Based on GSE86099, GSE161533, and TCGA-ESCA

**Figure 1:**
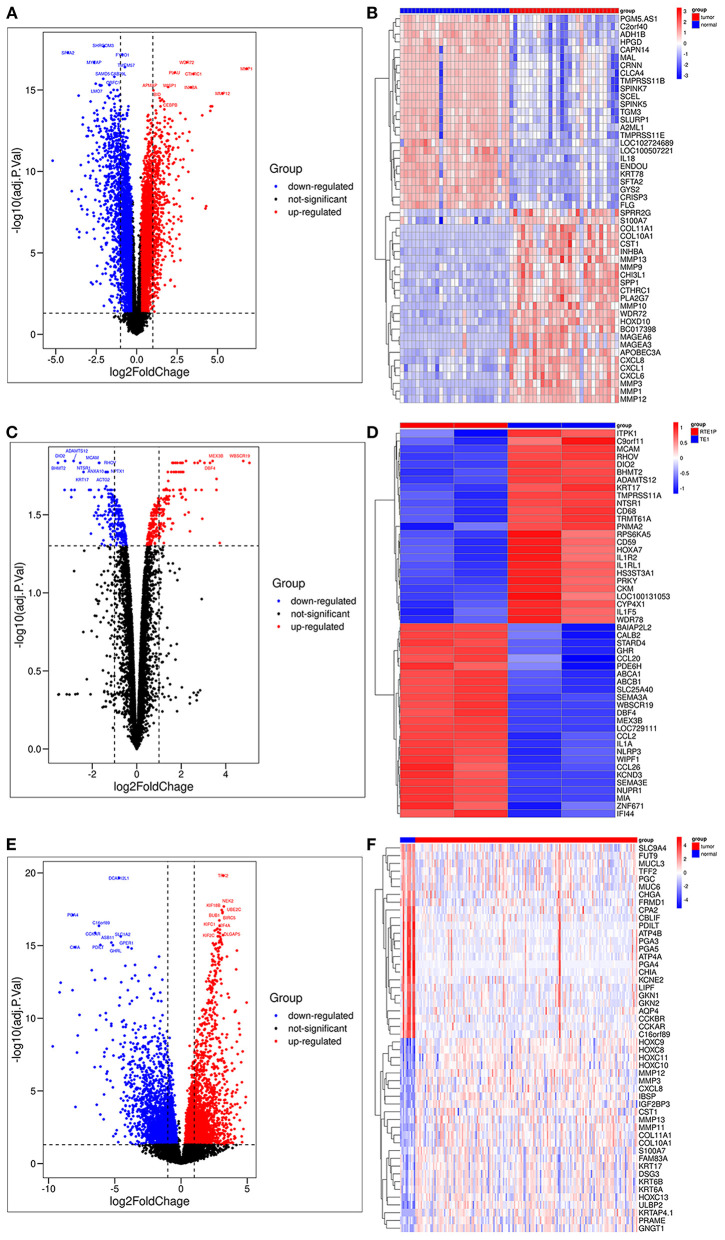
Volcano plot and heatmap of the differentially expressed genes in the GSE161533, GSE86099, and The Cancer Genome Atlas Esophageal Carcinoma (TCGA-ESCA) datasets. **(A)** Volcano plot of GSE161533 with the top 10 significant genes in the upregulated and downregulated clusters (ranked by *p*_adj._ value) highlighted. **(B)** Expression levels of the top 25 significant genes in the upregulated and downregulated clusters (ranked by |log2FoldChange|) of GSE161533. **(C)** Volcano plot of GSE86099 with the top 10 significant genes in the upregulated and downregulated clusters (ranked by *p*_adj._ value) highlighted. **(D)** Expression levels of the top 25 significant genes in the upregulated and downregulated clusters (ranked by |log2FoldChange|) of GSE86099. **(E)** Volcano plot of TCGA-ESCA with the top 10 significant genes in the upregulated and downregulated clusters (ranked by *p*_adj._ value) highlighted. **(F)** Expression levels of the top 25 significant genes in the upregulated and downregulated clusters (ranked by |log2FoldChange|) of TCGA-ESCA.

A total of 7,460 DEGs in tumor and normal tissues from GSE161533 are shown in Figure 1A, with 3,933 upregulated and 3,527 downregulated genes (*p*_adj._ < 0.05, |log2FoldChange| > 0.263). The top 25 DEGs in two clusters (upregulated or downregulated) are shown in Figure 1B (ranked by |log2FoldChange|). As shown in Figure 1C, 548 genes were significantly differentially expressed between the paclitaxel resistance cell lines and the non-paclitaxel resistance cell lines (*p*_adj._ < 0.05, |log2FoldChange| > 0.263) in GSE86099. Among them, 275 genes were upregulated and 273 genes were downregulated. The expressions of the top 25 DEGs in the upregulated and downregulated clusters are shown in Figure 1D. A total of 5,842 DEGs (2,963 upregulated and 2,879 downregulated) were found in the tumor group compared with the normal group from TCGA-ESCA; the cutoff values were *p*_adj._ < 0.05 and |log2FoldChange| > 0.263. The volcano plot and heatmap of the DEGs in TCGA-ESCA are shown in Figures 1E,F, respectively.

### WGCNA and Identification of the Key Modules

**Figure 2:**
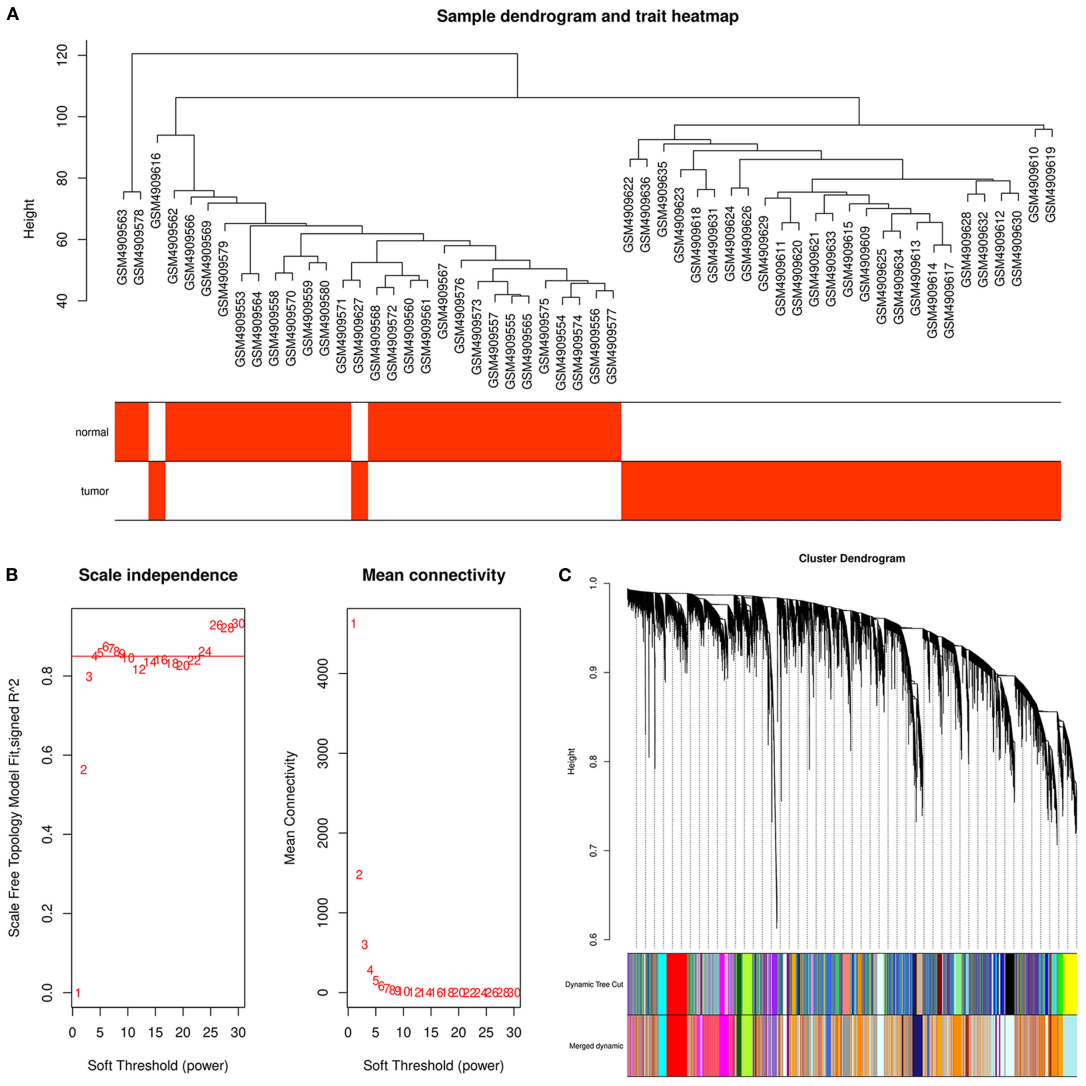
Construction of the weighted gene co-expression network based on GSE161533. **(A)** Sample dendrogram and trait heatmap. *Red* or *white bar codes underneath the dendrogram* correspond to the presence or absence of a pair of mutually exclusive clinical traits. **(B)** Identification of soft-thresholding powers based on scale independence and mean connectivity. The minimum power that satisfied a scale-free fit index of 0.8 was selected. **(C)** Module identification using dynamic tree cut. The minimum module size was 30. Modules with the top 25% similarity were merged.

**Figure 3:**
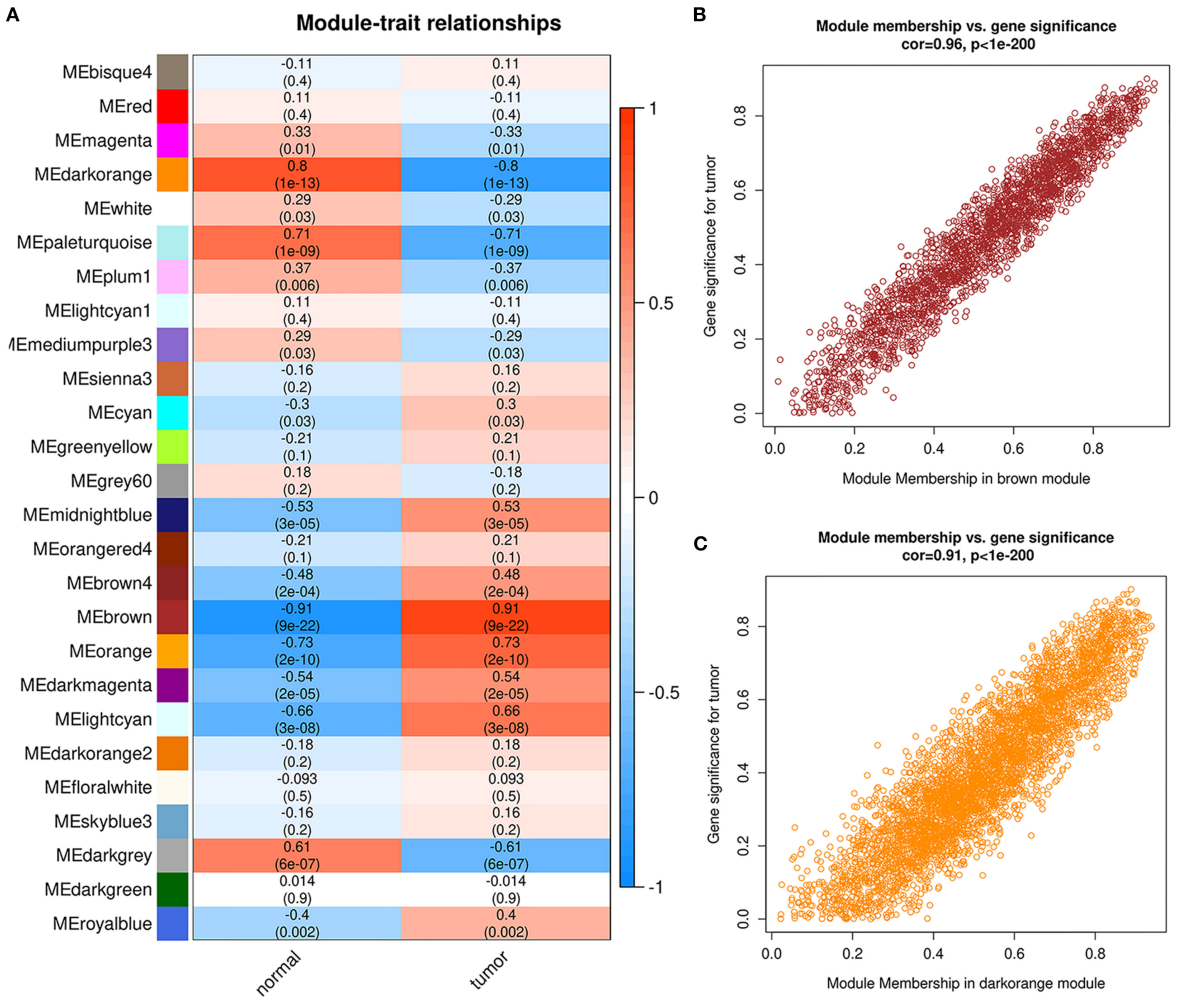
Identification of esophageal squamous cell carcinoma (ESCC)-specific modules of GSE161533. **(A)** Heatmap of module–trait relationships. **(B,C)** Scatter plots of module membership vs. gene significance in the brown **(B)** and dark orange **(C)** modules.

WGCNA was used to identify disease-associated modules wherein genes exhibited coordinated expression patterns, which greatly improved the chance of identifying hub genes. The sample dendrogram and trait heatmap of the GSE161533 dataset are shown in Figure 2A. As shown in Figure 2B, power of β = 4 was selected to conduct further analysis. As depicted in Figure 2C, by setting the minModuleSize to 30 (relatively large modules would be detected), numerous modules were identified by dynamic clustering; these modules were further merged based on their similarity by setting the MEDissThres to 0.25 (modules with the top 25% similarity were merged). As a result, a total of 26 merged modules were generated. Finally, we chose the modules with the highest correlations with the external trait “ESCC tumor” (positive and negative correlations) as the ESCC-associated modules (Figure 3A), which were a brown module (cor = 0.91, *p* = 9e−22) and a dark orange module (cor = −0.8, *p* = 1e−13). The correlations between module membership and gene significance of the ESCC-associated modules are shown in Figures 3B,C. Corresponding information of the ESCC-associated modules are provided in Supplementary Table 1.

### Identification of Paclitaxel Resistance- and Non-paclitaxel Resistance-Specific Genes

**Figure 4:**
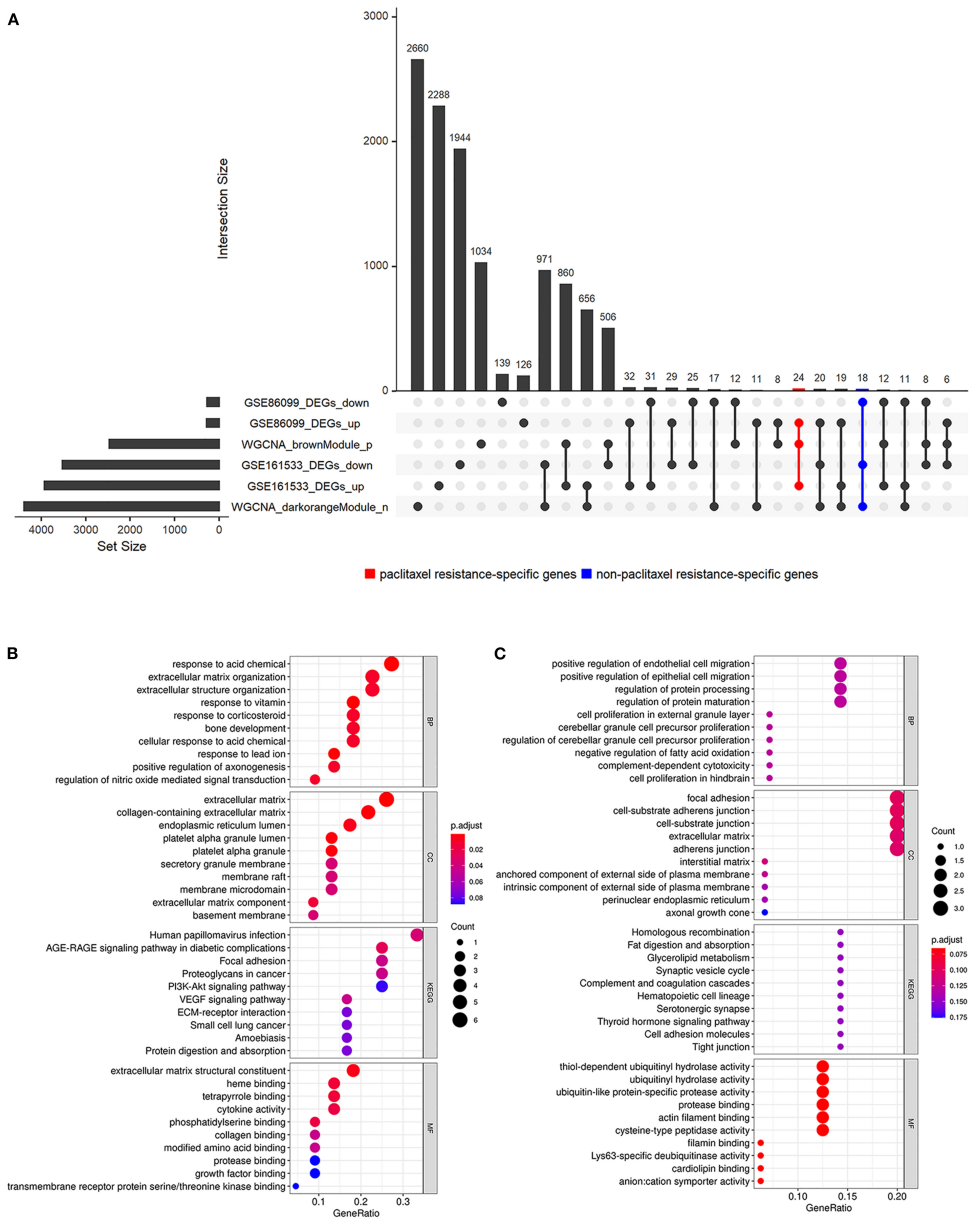
Intersection and functional enrichment analysis of the differentially expressed genes (DEGs) of two Gene Expression Omnibus (GEO) datasets and the genes in esophageal squamous cell carcinoma (ESCC)-specific modules. **(A)** Upset plot of the DEGs identified in GSE86099 and GSE161533 and the genes in the ESCC-specific modules selected by weighted gene co-expression network analysis (WGCNA). **(B,C)** Function enrichment analyses of the paclitaxel resistance-specific **(B)** and non-paclitaxel resistance-specific **(C)** genes.

We analyzed the intersection of the DEGs obtained from GSE86099 and GSE161533 and the genes in two ESCC-associated modules. As shown in Figure 4A, a total of 24 genes (*INHBA, MLLT11, PTGS2, PHTF2, CCL26, FN1, MFAP2, SPARC, MME, FKBP14, SHOX2, NUAK1, CYP26B1, MUCL1, ASAP1, KDELC1, TSPAN9, VEGFA, COL1A1, HTRA1, GUCY1A2, OLR1, KIF3C*, and *CLDN1*) with high expression in ESCC tumor, high expression in paclitaxel resistance, and positively associated with ESCC tumor status were selected as paclitaxel resistance-specific genes. The biological function and pathways (Figure 4B) of the paclitaxel resistance-specific genes were enriched in the response to acid chemical, extracellular matrix organization, collagen-containing extracellular matrix, endoplasmic reticulum lumen, extracellular matrix structural constituent, human papillomavirus infection, and focal adhesion.

Similarly, a total of 18 non-paclitaxel resistance-specific genes (*DIO2, PLEKHN1, DGAT2, CD59, CCBE1, USP43, ZBED2, SLC6A4, BRCC3, ZFYVE21, L1CAM, SQRDL, NEBL, AMOTL1, ARNTL2, TMEM45B, LRRC20*, and *ADAMTSL4*) were obtained from the intersection of the genes with low expression in ESCC tumor samples, low expression in the paclitaxel resistance cell lines, and negatively associated with ESCC tumor status (Figure 4A). The GO and KEGG analyses (Figure 4C) demonstrated that the non-paclitaxel resistance-specific genes were involved in the pathways of positive regulation of endothelial cell migration, focal adhesion, cell–substrate junction, thiol-dependent ubiquitinyl hydrolase activity, homologous recombination, fat digestion, and absorption.

### LASSO Analysis and Expression Levels of Key Genes

**Figure 5:**
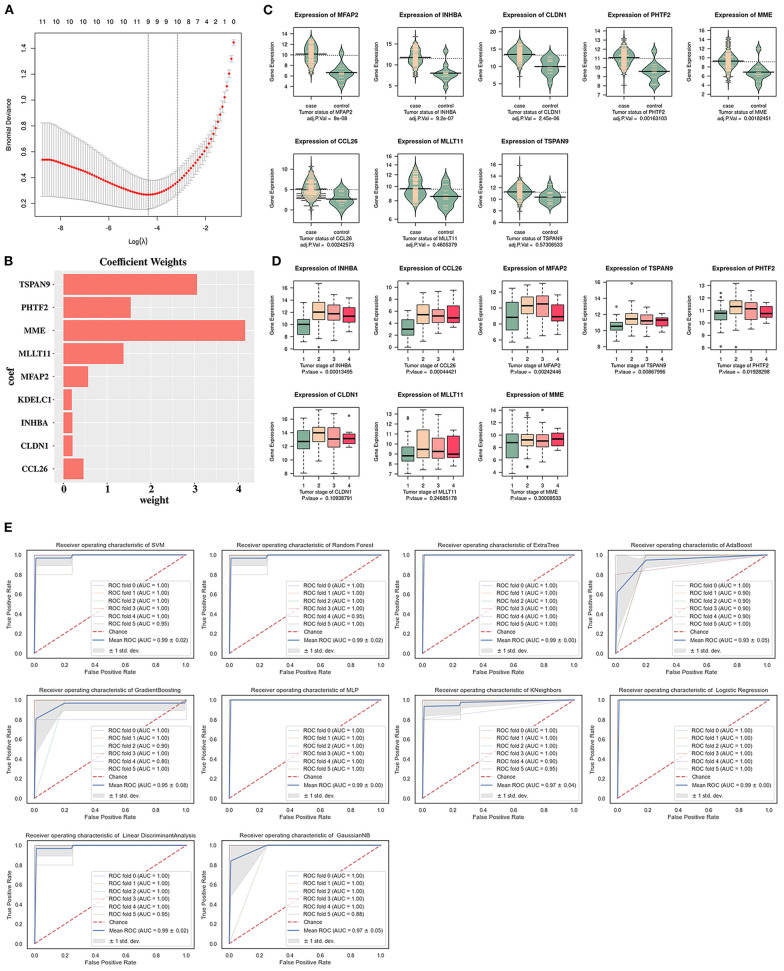
Identification of the paclitaxel resistance-specific key genes by least absolute shrinkage and selection operator (LASSO) analysis based on GSE161533. **(A)** Cross-validation of LASSO analysis of the paclitaxel resistance-specific key genes. **(B)** Coefficient weights of the paclitaxel resistance-specific key genes. **(C)** Expression levels of the paclitaxel resistance-specific key genes in the tumor group compared with the normal group based on The Cancer Genome Atlas Esophageal Carcinoma (TCGA-ESCA). **(D)** Expression levels of the paclitaxel resistance-specific key genes in four tumor stages based on TCGA-ESCA. **(E)** Area under the ROC curve (AUC) values of 10 esophageal squamous cell carcinoma (ESCC) classifiers of the paclitaxel resistance-specific key genes based on GSE161533.

For feature selection and machine learning validation, the samples were first grouped based on “tumor” and “normal” labels. According to the LASSO analysis (Figures 5A,B), nine paclitaxel resistance-specific key genes were obtained, namely, *PHTF2, MFAP2, MME, INHBA, TSPAN9, MLLT11, CLDN1, KDELC1*, and *CCL26*. Except for *KDELC1* (not found in TCGA-ESCA), *MLLT11*, and *TSPAN9*, the expression levels of the paclitaxel resistance-specific key genes were significant in the ESCC tumor group compared with the normal group (*p*_adj._ < 0.05) (Figure 5C). The expression levels of *PHTF2, MFAP2, INHBA, TSPAN9*, and *CCL26* (*p* < 0.05) displayed tumor stage-dependent alterations (Figure 5D). The AUC value was used to evaluate the classification performance of the ESCC machine classifiers based on the nine paclitaxel resistance-specific key genes. The AUCs of all the ESCC machine classifiers reached 0.95, except for the classifiers based on the AdaBoost algorithm (Figure 5E).

**Figure 6:**
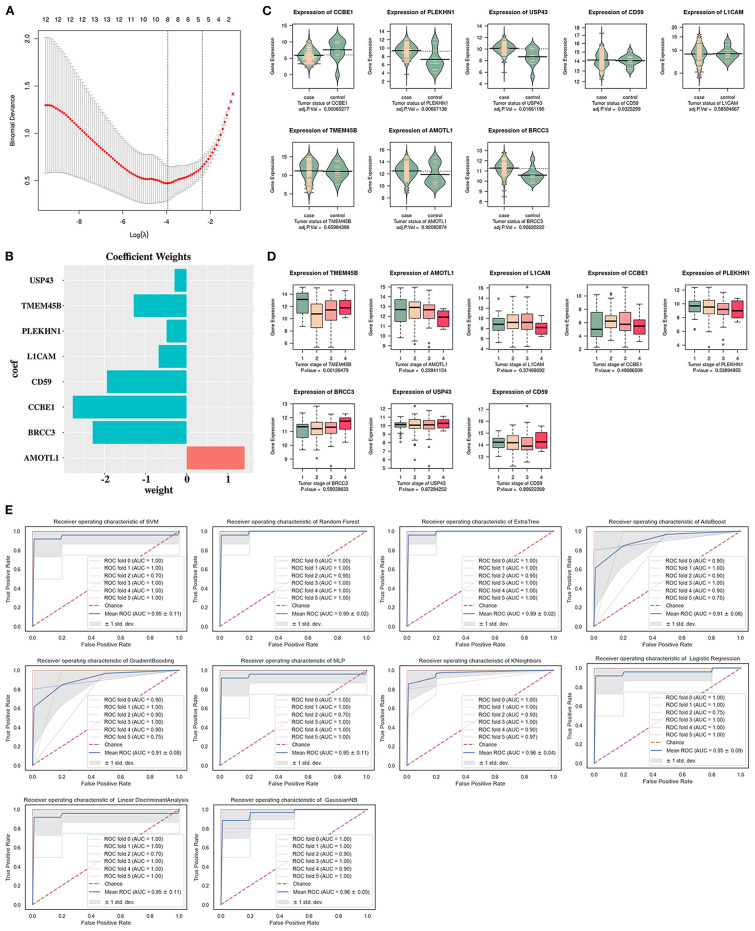
Identification of the non-paclitaxel resistance-specific key genes by least absolute shrinkage and selection operator (LASSO) analysis based on GSE161533. **(A)** Cross-validation of LASSO analysis of the non-paclitaxel resistance-specific key genes. **(B)** Coefficient weights of the non-paclitaxel resistance-specific key genes. **(C)** Expression levels of the non-paclitaxel resistance-specific key genes in the tumor group compared with the normal group based on The Cancer Genome Atlas Esophageal Carcinoma (TCGA-ESCA). **(D)** Expression levels of the non-paclitaxel resistance-specific key genes in four tumor stages based on TCGA-ESCA. **(E)** Area under the ROC curve (AUC) values of 10 esophageal squamous cell carcinoma (ESCC) classifiers of the non-paclitaxel resistance-specific key genes based on GSE161533.

As shown in Figures 6A,B, a total of eight key genes associated with non-paclitaxel resistance were obtained by LASSO analysis: *CD59, L1CAM, BRCC3, PLEKHN1, AMOTL1, TMEM45B, CCBE1*, and *USP43*. The expression levels of *CCBE1, PLEKHN1*, and *USP43* were significantly different in the ESCC tumor group compared with the normal group (Figure 6C). The expression level of *TMEM45B* displayed tumor stage-dependent alterations (*p* < 0.05) **(Figure 6D**). Except for the ESCC machine classifiers based on the AdaBoost and Gradient Boosting algorithms, the AUC values of the remaining ESCC machine classifiers were higher than 0.95 (Figure 6E).

In the independent dataset GSE164158, we verified the biological significance of the key genes using BPNN, as shown in Supplementary Figure 1, to classify the ESCC tumor samples from normal controls. The BPNN model based on nine paclitaxel resistance-specific key genes achieved an AUC value of 0.924 in the test set; the BPNN model based on eight non-paclitaxel resistance-specific key genes showed an inferior performance, with an AUC of 0.7046 (Supplementary Figure 2). These results corroborated the biological significance of the identified key genes. In addition, box plots in Supplementary Figures 1, 2 show that the majority of the paclitaxel resistance-specific key genes and the non-paclitaxel resistance-specific key genes displayed a consistent trend of tumor vs. normal expression differences across the training set (GSE161533) and the test set (GSE164158).

### PPI Network Analysis of Paclitaxel Resistance-Related Genes

**Figure 7:**
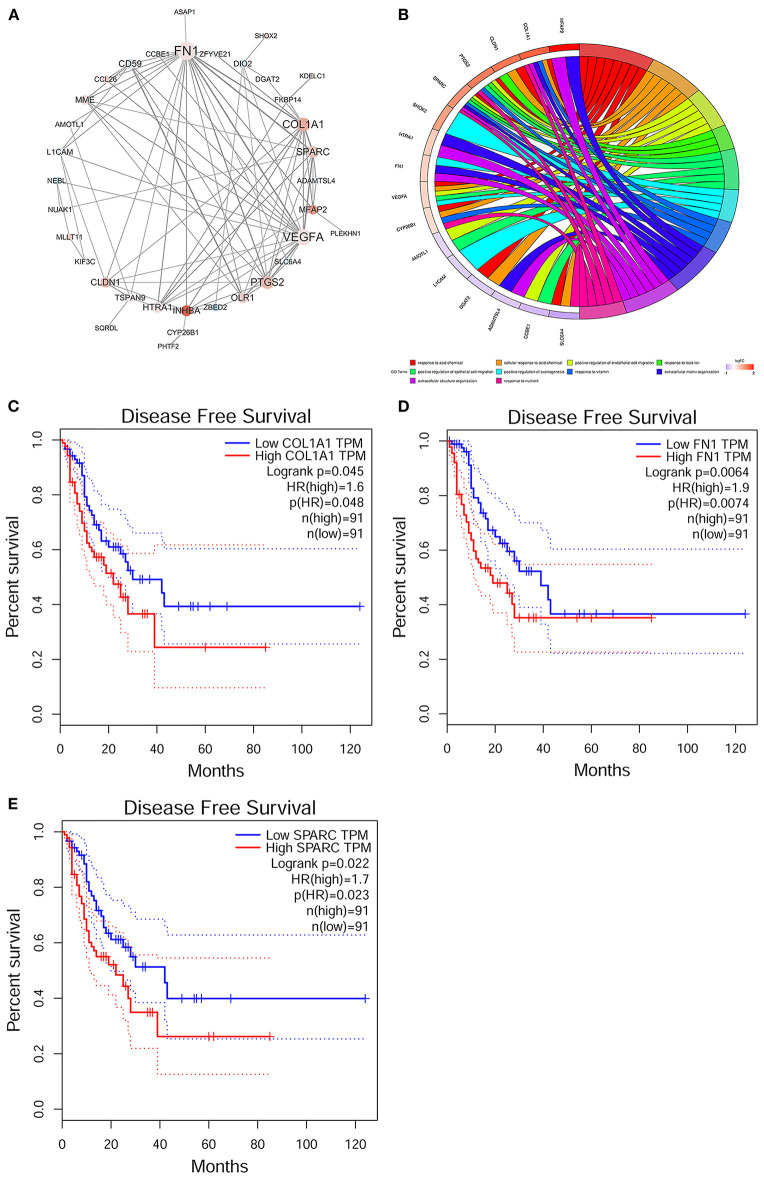
Protein–protein interaction (PPI) network analysis and survival analysis of the paclitaxel resistance-specific and non-paclitaxel resistance-specific genes. **(A)** PPI network. *Red points* indicate paclitaxel resistance-specific genes and *blue points* indicate non-paclitaxel resistance-specific genes. The size of the node was determined by the degree of each node and the color depth of the node determined by the |log2FoldChange| of the gene. The thickness of the node connection depends on the combined scores between the nodes. **(B)** The top 10 Gene Ontology (GO) terms and corresponding genes in the PPI network were visualized in the chord diagram. **(C–E)** Disease-free survival analyses of *COL1A1* **(C)**, *FN1* **(D)**, and *SPARC* **(E)** based on The Cancer Genome Atlas Esophageal Carcinoma (TCGA-ESCA).

The PPI network was used to discover the interactions of 24 paclitaxel resistance-specific genes and 18 non-paclitaxel resistance-specific genes. As shown in Figure 7A, a total of 35 nodes and 85 edges were found in the PPI network. Five hub genes (*FN1, VEGFA, COL1A1, PTGS2*, and *SPARC*) were selected with the degree of nodes larger than eight. FN1 has the greatest number of connections within the network, implying that this gene plays a central role in the network. The top 10 GO terms (Figure 7B) of the nodes in the PPI network suggested that these genes were enriched in response to acid chemical, cellular response to acid chemical, positive regulation of endothelial cell migration, response to lead ion, positive regulation of epithelial cell migration, positive regulation of axonogenesis, response to vitamin, extracellular structure organization, and response to nutrient. The most representative genes involved in the 10 GO terms were *MFAP2, COL1A1, CLDN1, PTGS2, SPARC, SHOX2, HTRA1, FN1, VEGFA, CYP26B1, AMOTL1, L1CAM, DGAT2, ADAMTSL4, CCBE1*, and *SLC6A4*. Among them, *SPARC, PTGS2, VEGFA*, and *COL1A1* participated in the greatest number of GO terms, and these genes were all associated with paclitaxel resistance. The survival analysis (Figures 7C–E) demonstrated that high expression levels of *COL1A1, FN1*, and *SPARC* were associated with a poor prognosis of ESCC patients (*p* < 0.05).

### Drug Interaction Prediction for Paclitaxel Resistance-Specific Key Genes

The relationship between the paclitaxel resistance-specific genes and the corresponding potential therapeutic candidates was retrieved from DGIdb. As presented in Supplementary Table 2, a total of 207 drugs were predicted to interact with paclitaxel resistance-specific genes. Among them, the drugs with the highest number of target genes were bevacizumab, capecitabine, celecoxib, lenalidomide, naproxcinod, ocriplasmin, oxaliplatin, and ranibizumab.

## Discussion

As a subtype of esophageal cancer (EC), ESCC is highly invasive. In the current study, a total of 548 and 7,460 DEGs were identified from GSE86099 and GSE161533, respectively. Based on GSE161533, WGCNA was used to construct a co-expression matrix, where 26 modules were obtained, among which two ESCC-specific modules were selected for subsequent analyses. Combining the DEGs identified in the above-mentioned two GEO datasets and the genes in the ESCC-specific modules, a total of 24 paclitaxel resistance-specific genes and 18 non-paclitaxel resistance-specific genes were identified. LASSO was used to identify the key genes associated with paclitaxel resistance and those associated with non-paclitaxel resistance with expression levels across different ESCC tumor stages, which were further confirmed in the TCGA-ESCA dataset. Additionally, 10 machine learning algorithms were used to construct ESCC classifiers based on the expression data of the key genes. Next, a PPI network was constructed to visualize the interactions between the paclitaxel resistance-specific genes. The nodes with a degree higher than 8 in the PPI network were selected as the hub genes; the survival analysis showed that high expressions of *COL1A1, FN1*, and *SPARC* indicate poor prognosis. Additionally, paclitaxel resistance-specific key genes were used to predict drugs that may be effective in paclitaxel-resistant ESCC. Our results showed that the paclitaxel resistance-specific key genes might be potential target sites for bevacizumab, capecitabine, celecoxib, ranibizumab, and abt-510.

PI3K, mTOR, and AKT were reported to be inhibited by paclitaxel at both the expression and phosphorylation levels (Xu et al., 2020), while our current function enrichment analyses showed that the paclitaxel resistance-specific genes were involved in the PI3K–Akt signaling pathway, implying that paclitaxel resistance-specific genes might prevent the PI3K–Akt signaling pathway from being inhibited by paclitaxel. In addition, aberrant hyperplasia of the extracellular matrix (ECM) was proposed to be associated with chemotherapy resistance, which agreed with our current results showing the enrichment of paclitaxel resistance-specific genes in the ECM–receptor interaction pathway (Zhou et al., 2020).

We confirmed the key genes significantly associated with the prognosis of ESCC tumor status through LASSO analysis and machine learning approaches. A total of nine key genes associated with paclitaxel resistance were obtained by using LASSO, in which the expression levels of *MFAP2, MME, INHBA, CLDN1, PHTF2*, and *CCL26* were upregulated in the tumor group based on TCGA-ESCA, which is consistent with the previous reports discussed above. Moreover, significant differences were found in the expressions of *INHBA, CCL26, MFAP2, TSPAN9*, and *PHTF2* in tumor stage based on TCGA-ESCA, suggesting their prognostic value in ESCC tumor development. Machine learning has been widely applied in cancer prognosis and prediction. A recent study has reported a support vector machine (SVM) classifier based on 75 features that can be used to predict the prognosis of ESCC patients (Yu et al., 2020). The SVM based on clinicopathological parameters together with 14-3-3σ expression generated an AUC in the validation cohort of 0.82 (Qi et al., 2014). In this study, the AUC values of the ESCC machine classifiers based on the key genes associated with paclitaxel resistance reached 0.9 and had prognostic potential in identifying patients with ESCC. We suggest that the ESCC machine classifiers may also work in the determination of sensitivity to paclitaxel in ESCC patients. Additionally, we also validated the prognostic value of the key genes associated with non-paclitaxel resistance, which showed prognostic potential as well.

To investigate the interactions between the ESCC-specific genes, we constructed a PPI network. In this network, we found that the five nodes with the highest degree centrality were *FN1, VEGFA, COL1A1, PTGS2*, and *SPARC*, suggesting that these genes were key signatures driving carcinogenesis and paclitaxel resistance in ESCC. The Kaplan–Meier survival analysis suggested good prognosis in ESCC patients with low expressions of *COL1A1, SPARC*, and *FN1*. In order to uncover the key pathway of paclitaxel resistance in ESCC, we investigated the biological function and pathways of the ESCC-specific genes in the PPI networks. The result was in line with a previous study showing a high expression of *FN1* in ESCC (Li et al., 2020). In EC, the upregulation of *FN1* expression is regulated by *STAB1* (Song et al., 2017). A study also reported that the suppression of *LTBP1* can attenuate cancer-associated fibroblast (CAF) transformation and inhibit *FN1* in ESCC (Cai et al., 2020). The elevated *FN1* expression may also promote the occurrence of cancer, including breast cancer (Dorman et al., 2016). However, there is no research report on how *FN1* promotes the formation of paclitaxel resistance in ESCC. We speculate that *FN1* may participate in the paclitaxel resistance of ESCC through the response to nutrient, extracellular matrix organization, and positive regulation of epithelial cell migration, which can explain the association between *FN1* and cell migration (Steffens et al., 2012). *SPARC* can serve as a therapeutic target in ESCC since the high expression of *SPARC* can predict tumor prognosis (Chen et al., 2017), which was consistent with our findings. Downregulating the expression of *SPARC* can reduce the migration and invasion of tumor cells in ESCC (Zhang et al., 2020). A previous study demonstrated that the overexpression of *SPARC* may be associated with response to nanoparticle albumin-bound paclitaxel (nab-paclitaxel in neck cancer) (Desai et al., 2009). Similarly, *SPARC* can be used to predict the response to nanoparticle-bound paclitaxel (nab-paclitaxel) in non-small cell lung cancer (NSCLC) (Komiya et al., 2016). Herein, we found that *SPARC* may be involved in the regulation of paclitaxel resistance in ESCC by extracellular matrix organization and positive regulation of epithelial cell migration. *COL1A1* is a type of collagen (COL) that has been reported to be upregulated in ESCC (Li et al., 2019) and may contribute to paclitaxel and topotecan resistance in ovarian cancer cells (Januchowski et al., 2016). It is reported that *COL1A1* is the target of miR-29, and the downregulation of miR-29 can promote the cisplatin resistance of ovarian cancer cells (Yu et al., 2014). To date, limited studies have reported on the relationship between *COL1A1* and paclitaxel sensitivity in ESCC. Based on the results of functional enrichment analysis, we speculate that *COL1A1* may regulate the sensitivity of ESCC to paclitaxel through ECM-related pathways, which is corroborated by the findings that the α1 chain coded by *COL1A1* is an essential component of the ECM (Rousseau et al., 2014).

Furthermore, we predicted the drugs regulating paclitaxel sensitivity in ESCC patients. The DGIdb can provide data on the interaction between drugs and paclitaxel resistance-specific genes based on existing resources. Among the 207 predicted drugs, some have been reported in ESCC treatment. Bevacizumab has already been used in chemotherapy in ESCC, which targeted vascular endothelial growth factor A (VEGF-A) (Yang et al., 2020). Paclitaxel plus bevacizumab is reported as a method of treatment for HER2-negative metastatic breast cancer, which had a better effect than paclitaxel treatment alone (Delaloge et al., 2016). Recently, a study has suggested that the addition of bevacizumab may contribute to the treatment of non-squamous NSCLC (Cortot et al., 2020). Thus, the combination of bevacizumab and paclitaxel in ESCC treatment may reduce the paclitaxel resistance of patients. Celecoxib is the inhibitor of cyclooxygenase-2 (COX-2) and was reported to enhance the antitumor effects of chemotherapy and radiotherapy for ESCC (Yusup et al., 2014; Kim and Shah, 2017). Our study showed the treatment ability of celecoxib in ESCC and was supported by a previous study, demonstrating that celecoxib may exert antitumor effects by blocking the blood flow to the tumor cell. We predicted that capecitabine might target paclitaxel resistance-specific genes, and the findings were in line with a previous study demonstrating that capecitabine might be a therapeutic candidate for ESCC. It was reported that the cell viability was significantly reduced with paclitaxel and celecoxib combination therapy in ovarian cancer (Kim et al., 2014). Another study revealed the effect of celecoxib and taxol on multidrug resistance in human breast cancer (Liu et al., 2011), which can be an alternative treatment method for paclitaxel-resistant ESCC. Although we have only discussed some of the predicted drugs, other candidates also deserve further investigation in the treatment of paclitaxel-resistant ESCC.

We found several genes that were significantly related to ESCC tumor status and constructed ESCC machine classifiers to determine their prognostic potential in ESCC. Through PPI analysis, we revealed the genes and possible pathways associated with paclitaxel resistance in ESCC. Finally, we used the database to predict drugs related to paclitaxel resistance. However, our results are mainly based on public data and existing reports, lacking experimental proof. Due to limited samples, we were unable to verify the genes related to paclitaxel resistance in ESCC in other datasets or at the animal level. Nevertheless, we plan to further verify our current findings in mouse models.

In summary, our study identified paclitaxel resistance-specific genes, along with their predicted pathways and biological functions. Based on LASSO analysis and machine learning, some of these genes were confirmed as good predictors of ESCC patients' survival. The predicted drugs have the potential to be used in combination with paclitaxel to reduce paclitaxel resistance in ESCC patients and improve therapeutic effectiveness. These findings may help in understanding the mechanisms of drug resistance and in discovering potential targets to overcome paclitaxel resistance, which may help improve the therapeutic outcomes of ESCC patients.

## Data Availability Statement

Publicly available datasets were analyzed in this study. This data can be found at: GSE67269, GSE86099, and GSE161533 from TCGA database.

## Author Contributions

ZS, MC, FL, HX, PZ, and JL contributed to the drafting of the manuscript and data analysis and revised the manuscript in accordance with reviewers' comments. ZS, MC, MK, and JL contributed to the study design and in reviewing the submitted manuscript. All the authors have read and approved the final version of the submitted manuscript.

## Conflict of Interest

The authors declare that the research was conducted in the absence of any commercial or financial relationships that could be construed as a potential conflict of interest.

## Publisher's Note

All claims expressed in this article are solely those of the authors and do not necessarily represent those of their affiliated organizations, or those of the publisher, the editors and the reviewers. Any product that may be evaluated in this article, or claim that may be made by its manufacturer, is not guaranteed or endorsed by the publisher.

## Supplementary Material

Supplementary Figure 1 ROC curve of BPNN prediction based on the nine paclitaxel resistance-specific key genes and box plots showing the expression profiles of these genes across training set and test set.

Supplementary Figure 2 ROC curve of BPNN prediction in based on the eight non-paclitaxel resistance-specific key genes and box plots showing the expression profiles of these genes across training set and test set.

Supplementary Table 1 The information of ESCC-specific modules identified by WGCNA.

Supplementary Table 2 The information of drug prediction of key genes associated with paclitaxel resistance.

## Abbreviations

ESCC: esophageal squamous cell carcinoma
DEG: differentially expressed gene
WGCNA: weighted gene co-expression network analysis
LASSO: least absolute shrinkage and selection operator
FDA: United States Food and Drug Administration
ECM: extracellular matrix
PPI: protein–protein interaction
GO: Gene Ontology
MM: module eigengene
PCC: Pearson's correlation coefficient
KEGG: Kyoto Encyclopedia of Genes and Genomes.
