# Supplementary figures and images for: Identification of Key Genes and Pathways Associated With Paclitaxel Resistance in Esophageal Squamous Cell Carcinoma Based on Bioinformatics Analysis
Source: Front Genet. 2021 Aug 11;12:671639. doi: 10.3389/fgene.2021.671639 (PMC8386171; doi:10.3389/fgene.2021.671639)

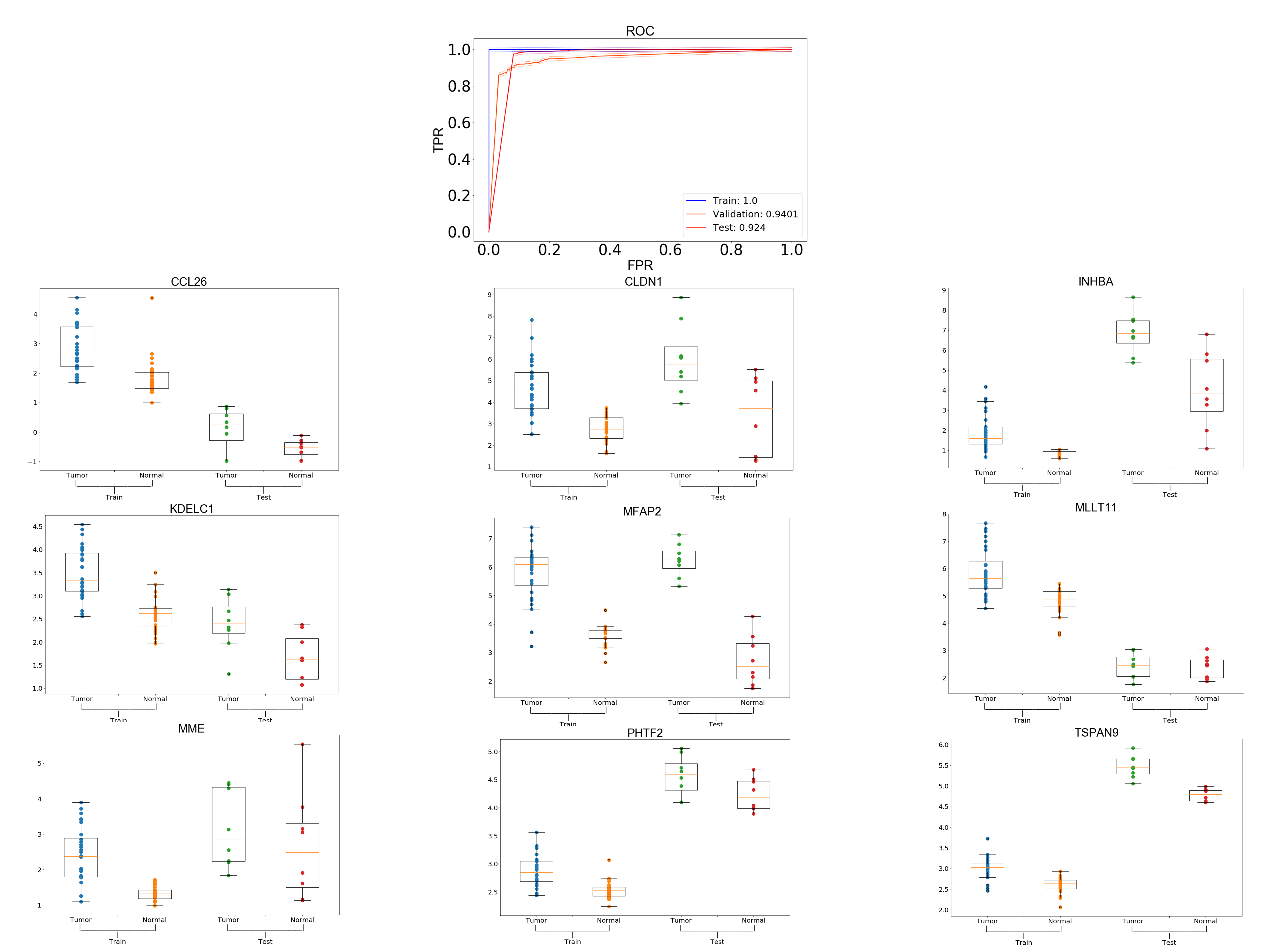

Supplement: Supplementary Figure 1 — ROC curve of BPNN prediction based on the nine paclitaxel resistance-specific key genes and box plots showing the expression profiles of these genes across training set and test set. [file Image_1.TIF]

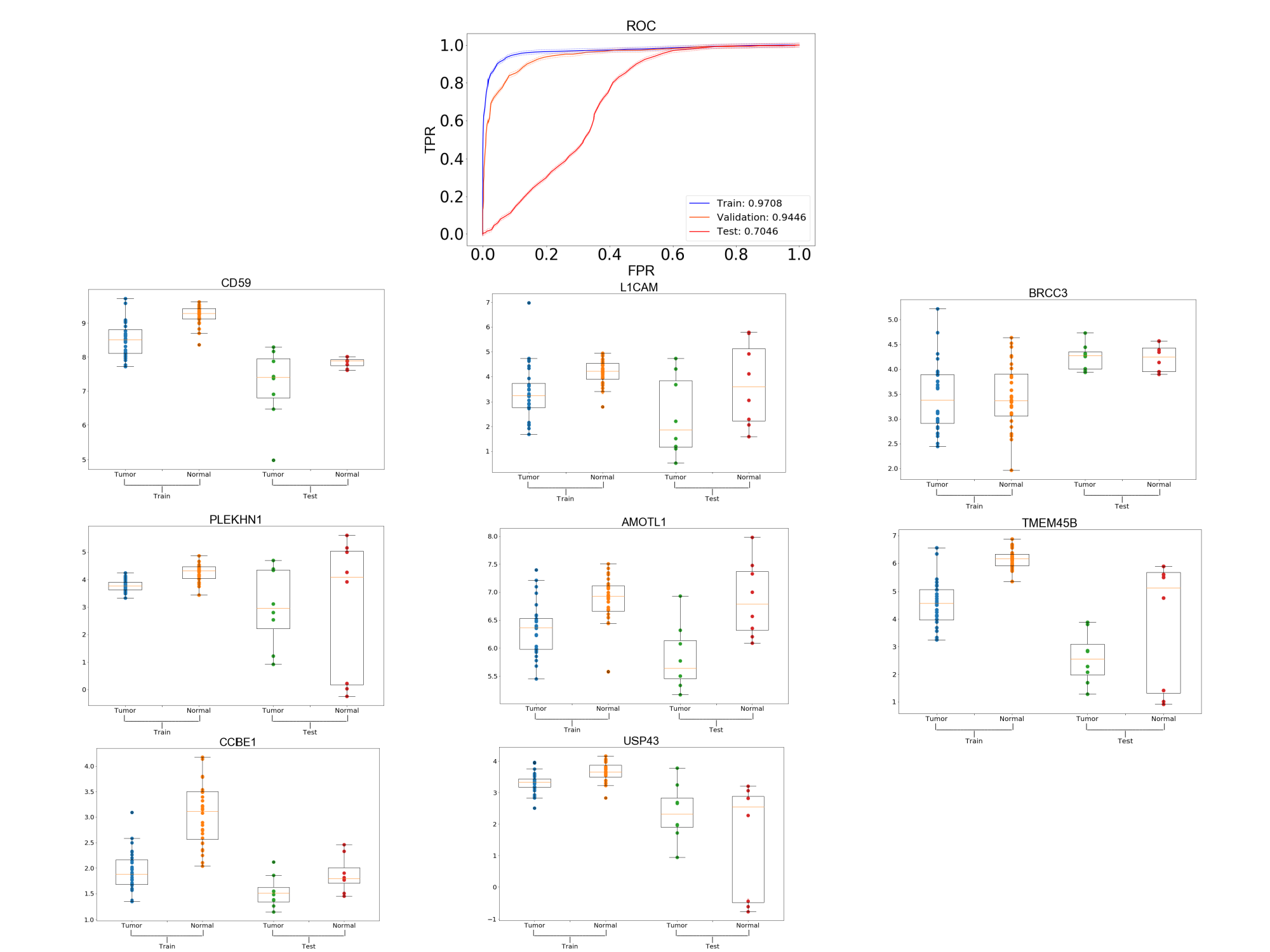

Supplement: Supplementary Figure 2 — ROC curve of BPNN prediction in based on the eight non-paclitaxel resistance-specific key genes and box plots showing the expression profiles of these genes across training set and test set. [file Image_2.TIF]
